# Safety signals of zolbetuximab in gastric or gastroesophageal junction adenocarcinoma: a comprehensive analysis of the FDA adverse event reporting system (FAERS) data

**DOI:** 10.3389/fonc.2026.1787423

**Published:** 2026-07-10

**Authors:** Li Lin, Maohua Chen, Chenmin Wu, Hong Chen

**Affiliations:** 1 Department of Medical Oncology, Shengli Clinical Medical College of Fujian Medical University, Fujian Provincial Hospital, Fuzhou University Affiliated Provincial Hospital, Fuzhou, China; 2 Department of Pharmacy, Pingtan Comprehensive Experimental Area Hospital, Fuzhou, China; 3 Department of Rheumatology and Immunology, Shengli Clinical Medical College of Fujian Medical University, Fujian Provincial Hospital, Fuzhou University Affiliated Provincial Hospital, Fuzhou, China; 4 Department of Neurology, Shengli Clinical Medical College of Fujian Medical University, Fujian Provincial Hospital, Fuzhou, China

**Keywords:** adverse event, disproportionality analysis, FAERS database, pharmacovigilance, zolbetuximab

## Abstract

**Background:**

Zolbetuximab is a novel monoclonal antibody targeting CLDN18.2 for advanced gastric or gastroesophageal junction adenocarcinoma. However, its large-scale, real-world safety profile remains incompletely characterized. This study aimed to analyze its post-marketing adverse events using the FDA Adverse Event Reporting System (FAERS).

**Methods:**

Adverse event reports from FAERS (Q1 2024 – Q3 2025) with zolbetuximab as the primary suspect were analyzed. Disproportionality analysis using four algorithms (ROR, PRR, IC, and EBGM) was performed to detect potential safety signals. Time-to-onset analysis was also conducted.

**Results:**

A total of 676 reports were analyzed. Disproportionality analysis confirmed known gastrointestinal and metabolic disorders while identifying several unlabeled adverse events, including but not limited to gastritis, protein-losing gastroenteropathy, peripheral edema, compression fracture, and vascular pain. The median time-to-onset was 0 days, with 82.9% of events occurring within the first 30 days.

**Conclusion:**

This first large-scale pharmacovigilance study of zolbetuximab detected several potential safety signals not currently listed in the prescribing information. These findings highlight the importance of post-marketing surveillance and should be interpreted as hypothesis-generating, warranting further validation in clinical and real-world studies.

## 1 Introduction

Gastric or gastroesophageal junction (G/GEJ) adenocarcinoma ranks as the fifth most common malignancy globally and is one of the leading causes of cancer-related mortality, with particularly high incidence and death rates in East Asia (1–3). Historically, the treatment of advanced G/GEJ adenocarcinoma has long relied on cytotoxic chemotherapy, yet patient outcomes remain generally poor (4). In recent years, with a deeper understanding of the molecular biological characteristics of tumors, the treatment paradigm for gastric cancer (GC) is shifting from traditional chemotherapy towards an era of precision medicine that combines targeted therapy and immunotherapy (5–8). Against this backdrop, the search for novel and highly specific therapeutic targets is key to breaking through the current treatment bottlenecks.

Zolbetuximab is a murine/human chimeric IgG1 monoclonal antibody that targets Claudin 18.2 (CLDN18.2) (9–11). Its target, CLDN18.2, is a tight junction protein. Under normal physiological conditions, the expression of CLDN18.2 is strictly confined to the tight junction complexes of differentiated gastric mucosal epithelial cells. Due to cellular polarity, it remains inaccessible to circulating antibodies (12–14). However, during the pathogenesis and progression of malignant tumors, such as G/GEJ adenocarcinoma, cellular polarity is disrupted. This disruption exposes the CLDN18.2 epitope on the surface of tumor cells, rendering it an ideal target for therapeutic agents (12, 15). Zolbetuximab specifically binds to CLDN18.2 on the surface of tumor cells via its Fab fragment. Subsequently, its Fc segment activates the immune system, primarily mediating the killing of tumor cells through two mechanisms: Antibody-Dependent Cellular Cytotoxicity (ADCC) and Complement-Dependent Cytotoxicity (CDC) (11, 14, 16, 17).

Preclinical studies have demonstrated that zolbetuximab exhibits antitumor activity in both xenograft and syngeneic mouse models of G/GEJ cancer, either as a monotherapy or in combination with chemotherapy (11). Furthermore, research in animal models suggests that the combination of zolbetuximab with anti-PD-1 antibodies can inhibit tumor growth more effectively, providing a rationale for future combination therapy strategies (11). The success of zolbetuximab has initiated a new era in CLDN18.2-targeted therapy (18–20).

Despite its therapeutic promise, a significant gap exists in the large-scale, real-world evidence regarding the safety of zolbetuximab. To address this knowledge gap, we interrogated the FDA Adverse Event Reporting System (FAERS) database (2024 Q1–2025 Q3) to mine and analyze adverse event reports. The primary aim was to detect undiscovered or unlabeled adverse reaction signals through disproportionality analysis. The results were subsequently evaluated against the existing prescribing information to uncover potential safety concerns and inform clinical practice.

## 2 Methods

### 2.1 Data sources

Data for this analysis were extracted from the FDA FAERS database, a quarterly updated spontaneous reporting system. We retrieved all reports submitted between Q1–2024 and Q3–2025 from the FDA’s public portal (https://fis.fda.gov/extensions/FPD-QDE-FAERS/FPD-QDE-FAERS.html). Consistent with established pharmacovigilance methodology and our previous work (21), the raw data underwent a standardized cleaning and deduplication protocol to ensure the integrity of subsequent disproportionality analysis. From the cohort of 2,851,820 unique reports, we identified 676 cases where zolbetuximab was designated as the primary suspect drug.

### 2.2 Data extraction and descriptive analysis

Within the FAERS, all adverse events (AEs) are systematically coded according to the Medical Dictionary for Regulatory Activities (MedDRA), version 26.0. The MedDRA terminology follows a five-tier hierarchical structure: System Organ Class (SOC), High-Level Group Term (HLGT), High-Level Term (HLT), Preferred Term (PT), and Lowest-Level Term (LLT). This structure allows for the standardized categorization of each reported AE into its corresponding SOCs. Our investigation leveraged this systematic framework to identify and analyze AEs associated with zolbetuximab.

A notable feature of the FAERS database is its inclusion of diverse drug nomenclature, encompassing both FDA-approved names and commonly used designations. To ensure a comprehensive case capture, drug entries for zolbetuximab were retrieved from the DRUG file using its generic name and unified code (e.g., “VYLOY”). The RxNorm terminology system served as the reference standard for this drug mapping procedure, ensuring terminological consistency (22).

In FAERS reports, the initial assessment of drug-AE relationships is provided by the submitter. To strengthen the validity of our analysis, we specifically selected cases in which zolbetuximab was assigned the role of “Primary Suspect” (PS). This focused selection criterion enhances the specificity of signal detection and the robustness of subsequent causal association assessments.

### 2.3 Data mining

To detect significant associations between zolbetuximab and reported adverse events, we conducted a disproportionality analysis—a widely used data mining approach in spontaneous reporting systems that quantifies the strength of drug-event associations relative to the background reporting frequency (23). Our analysis integrated four distinct and validated statistical measures: the Reporting Odds Ratio (ROR), Proportional Reporting Ratio (PRR), Information Component (IC), and Empirical Bayesian Geometric Mean (EBGM) (24). A conservative approach to signal generation was implemented, requiring that an association meet the significance thresholds for all four algorithms to be considered a potential safety signal. This multi-method framework mitigates the limitations inherent in any single measure. The specific calculation formulas, alongside their respective decision thresholds, are cataloged in **Supplementary Table 1**, while the complete set of contingency tables used in these calculations is available for review in **Supplementary Table 2**.

### 2.4 Time-to-onset analysis

The time-to-onset (TTO) for each adverse event was calculated based on data extracted from the FAERS database, defined as the duration between the recorded start date of zolbetuximab treatment (START_DT) and the date the adverse event was documented (EVENT_DT). As the derived TTO data were typically non-normally distributed, the median value was utilized to represent the central tendency and typical latency period.

## 3 Results

### 3.1 General characteristics

**Figure 1:**
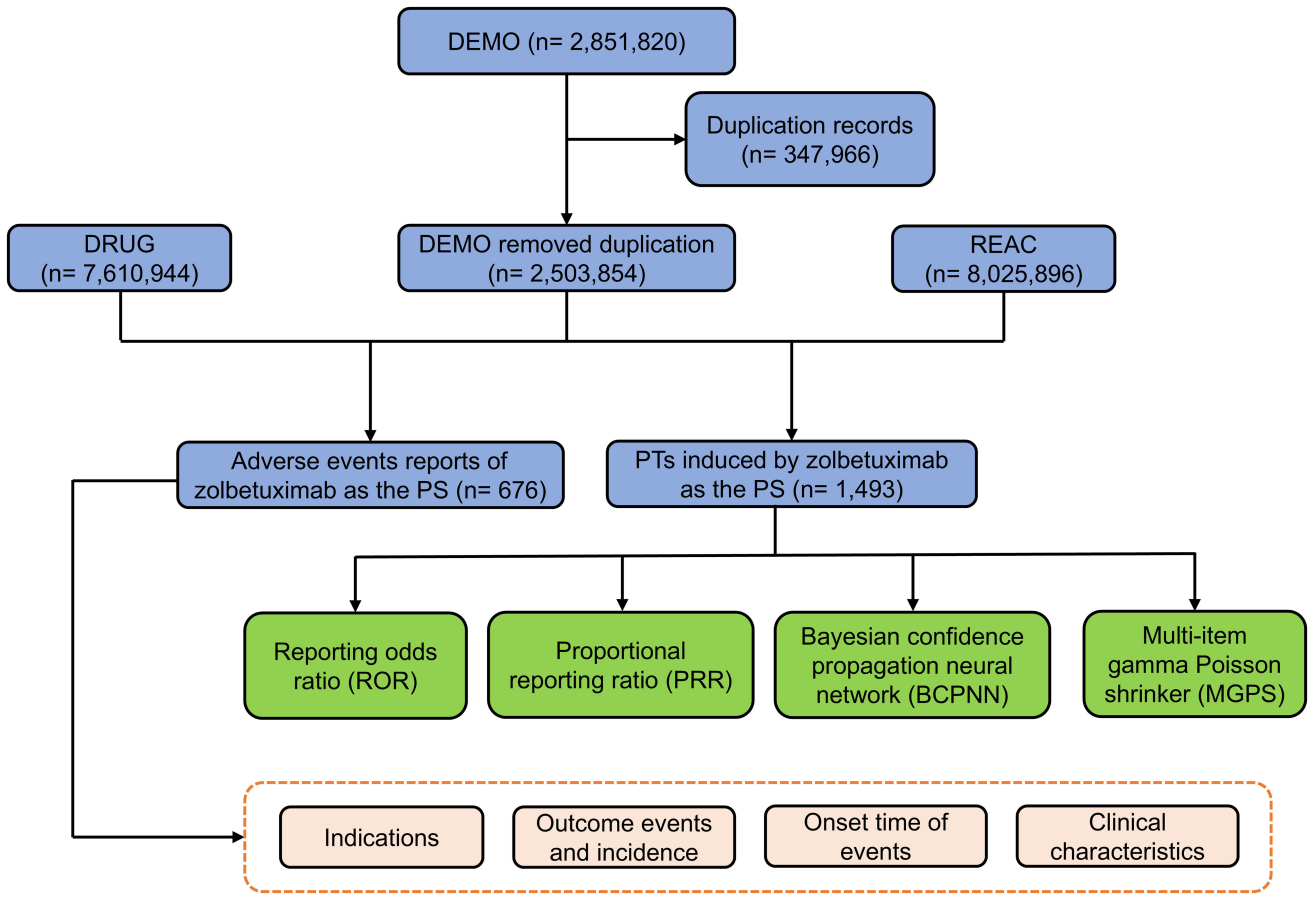
The flow diagram of selecting zolbetuximab-related AEs from FAERS database. DEMO, demographic file: DRUG, drug file; REAC, reaction file; PS, primary suspect.

As illustrated in **Figure 1**, a total of 2,851,820 individual adverse event reports were submitted to FAERS during the study period (January 2024 to September 2025). After applying stringent deduplication procedures and removing reports with critical missing information, a curated cohort of 676 zolbetuximab-associated reports was established for in-depth safety analysis. The study cohort comprised 676 reports, with demographic and clinical characteristics detailed in **Table 1**. **A** male predominance was observed (65.51%, n=414/632), while females accounted for (34.49%, n=218/632). The median patient age was 71 years, with elderly patients (≥65 years) representing the majority (71.04%, n=395/556) and adults aged 18–65 years comprising (28.42%, n=158/556). Geographically, most reports originated from Japan (70.56%, n=477/676). Gastric cancer was the predominant indication for zolbetuximab administration (81.37%, n=402/494). The most frequently reported combination drugs included oxaliplatin, capecitabine, and fluorouracil. Serious Outcome, encompassing death, life-threatening, hospitalization, disabling, and other serious outcomes, accounted for 83.58% of reports. The median TTO was 0 days (interquartile range: 0–17 days), suggesting a temporal relationship with drug initiation. Health professionals submitted the overwhelming majority of reports (98.66%, n=665/674). Temporal analysis revealed a marked increase in reporting frequency in 2025 (93.49%, n=632/676) compared to 2024 (6.51%, n=44/676).

**Table 1:** Clinical characteristics of reports with zolbetuximab from the FAERS database (January 2024 to September 2025).

Characteristics	Zolbetuximab-induced AE reports (n = 676)		
Number of events	Available number, n	Case number, n	Case proportion, %
Gender, n (%)	632	–	93.49%*
Female	–	218	34.49%
Male	–	414	65.51%
Age (years), n (%)	556	–	82.25%*
< 18	–	3	0.54%
18 ≤ and ≤ 65	–	158	28.42%
> 65	–	395	71.04%
Median (IQR)	–	71 (62 - 80)	–
Weight (Kg), n (%)	85	–	12.57%*
< 80	–	81	95.29%
80 ≤ and ≤ 100	–	3	3.53%
> 100	–	1	1.18%
Median (IQR)	–	56 (49 - 66)	–
Reported countries, n (%)	676	–	100.00%*
JP	–	477	70.56%
US	–	145	21.45%
FR	–	9	1.33%
Other country	–	45	6.66%
Indications, n (%)	494	–	73.08%*
Gastric cancer	–	364	73.68%
Gastric cancer stage iv	–	38	7.69%
Combination drugs, n (%)	464	–	68.64%*
Oxaliplatin	–	362	78.02%
Capecitabine	–	201	43.32%
Fluorouracil	–	174	37.50%
Outcomes, n (%)	676	–	100.00%*
Non-serious Outcome	–	111	16.42%
Serious Outcome	–	565	83.58%
Death	–	91	16.11%
Life-threatening	–	19	3.36%
Hospitalization	–	234	41.42%
Disability	–	7	1.24%
Other serious outcomes	–	407	72.04%
Time-to-onset (days)	193	–	28.55%*
Median (IQR)	–	0 (0-17)	–
Reporters, n (%)	674	–	99.70%*
Health professional	–	665	98.66%
Consumer	–	9	1.34%
Reporting year, n (%)	676	–	100.00%*
2024	–	44	6.51%
2025	–	632	93.49%

*Represents the proportion of the total cohort (n=676). All other percentages represent the proportion of their respective subgroups as indicated in the ‘Available Number’ column; IQR, interquartile range.

### 3.2 Disproportionality analysis

The systemic toxicity profile of zolbetuximab, as detected by disproportionality analysis at the SOC level, is summarized in **Table 2**. Pharmacovigilance mining revealed that zolbetuximab was associated with adverse events spanning 25 distinct organ systems. Among these, the SOCs with the most significant signal strengths and reporting frequencies were Gastrointestinal disorders (SOC: 10017947, n = 280), Metabolism and nutrition disorders (SOC: 10027433, n = 125), and Investigations (SOC: 10022891, n = 95).

**Table 2:** Signal strength of reports of zolbetuximab at the system organ class (SOC) level in FAERS database.

System organ class (SOC)	Zolbetuximab cases reporting SOC	ROR (95% two-sided CI)	PRR (χ2)	IC(IC025)	EBGM(EBGM05)
Metabolism and nutrition disorders	125	5.38 (4.47-6.49)^a^	4.89 (395.48)^a^	2.29 (1.90)^a^	4.89 (4.06)^a^
Gastrointestinal disorders	280	4.59 (4.00-5.25)^a^	3.68 (586.39)^a^	1.88 (1.64)^a^	3.68 (3.21)^a^
Neoplasms benign, malignant and unspecified (incl cysts and polyps)	69	3.14 (2.46-4.01)^a^	3.01 (94.42)^a^	1.59 (1.24)^a^	3.01 (2.36)^a^
Blood and lymphatic system disorders	51	2.30 (1.74-3.05)^a^	2.24 (35.73)^a^	1.16 (0.88)^a^	2.24 (1.69)
Investigations	95	1.71 (1.39-2.11)^a^	1.65 (25.80)	0.72 (0.59)^a^	1.65 (1.34)
Congenital, familial and genetic disorders	4	1.42 (0.53-3.79)	1.42 (0.49)	0.50 (0.19)^a^	1.42 (0.53)
Renal and urinary disorders	24	1.24 (0.83-1.86)	1.24 (1.12)	0.31 (0.21)^a^	1.24 (0.83)
Vascular disorders	29	1.11 (0.77-1.61)	1.11 (0.32)	0.15 (0.10)^a^	1.11 (0.77)
Respiratory, thoracic and mediastinal disorders	50	1.04 (0.78-1.38)	1.04 (0.07)	0.05 (0.04)^a^	1.04 (0.78)
Hepatobiliary disorders	11	0.89 (0.49-1.61)	0.89 (0.15)	-0.17 (-0.31)	0.89 (0.49)
Nervous system disorders	65	0.83 (0.65-1.07)	0.84 (2.02)	-0.25 (-0.32)	0.84 (0.66)
Endocrine disorders	3	0.72 (0.23-2.25)	0.73 (0.31)	-0.46 (-1.44)	0.73 (0.23)
Infections and infestations	44	0.65 (0.48-0.87)	0.66 (8.19)	-0.60 (-0.81)	0.66 (0.49)
General disorders and administration site conditions	125	0.61 (0.51-0.73)	0.65 (27.91)	-0.62 (-0.74)	0.65 (0.54)
Cardiac disorders	12	0.51 (0.29-0.90)	0.52 (5.53)	-0.95 (-1.68)	0.52 (0.29)
Skin and subcutaneous tissue disorders	25	0.41 (0.28-0.61)	0.42 (20.86)	-1.24 (-1.85)	0.42 (0.28)
Immune system disorders	7	0.39 (0.19-0.83)	0.40 (6.52)	-1.33 (-2.80)	0.40 (0.19)
Injury, poisoning and procedural complications	61	0.34 (0.26-0.43)	0.37 (75.99)	-1.43 (-1.85)	0.37 (0.29)
Eye disorders	6	0.26 (0.12-0.57)	0.26 (12.78)	-1.94 (-4.32)	0.26 (0.12)
Musculoskeletal and connective tissue disorders	11	0.23 (0.13-0.41)	0.24 (28.36)	-2.08 (-3.77)	0.24 (0.13)
Ear and labyrinth disorders	1	0.18 (0.03-1.27)	0.18 (3.79)	-2.48 (-17.65)	0.18 (0.03)
Psychiatric disorders	8	0.18 (0.09-0.35)	0.18 (30.88)	-2.47 (-4.94)	0.18 (0.09)
Reproductive system and breast disorders	1	0.13 (0.02-0.92)	0.13 (5.85)	-2.94 (-20.89)	0.13 (0.02)
Surgical and medical procedures	2	0.08 (0.02-0.31)	0.08 (22.21)	-3.67 (-14.71)	0.08 (0.02)
Product issues	1	0.03 (0.00-0.24)	0.03 (27.68)	-4.85 (-34.49)	0.03 (0.00)

^a^ indicates statistically significant signals in the algorithm.

ROR, reporting odds ratio; CI, confidence interval; PRR, proportional reporting ratio; χ2, chi-squared; IC, information component; IC025, the lower limit of 95% CI of the IC; EBGM, empirical Bayesian geometric mean; EBGM05, the lower limit of 95% CI of EBGM.

Signals for “Metabolism and nutrition disorders” and “Gastrointestinal disorders” were consistently positive across all four algorithms (ROR, PRR, IC, and EBGM). This finding demonstrates strong concordance with the drug’s approved label, which lists nausea, vomiting, decreased appetite, and weight loss as common adverse reactions, thereby validating the established safety profile. Similarly, a significant signal was detected for “Neoplasms benign, malignant and unspecified (incl cysts and polyps)” by all four methods, a finding that is primarily attributable to the underlying progression of the malignant disease itself in the treated patient population.

Disproportionality analysis at the SOC level revealed distinct signal patterns across various physiological systems. Significant associations were detected by three algorithms (ROR, PRR, and IC) for Blood and lymphatic system disorders (SOC: 10005329, n = 51). Signals identified by both ROR and IC algorithms were observed for Investigations (SOC: 10022891, n = 95). Furthermore, several SOCs generated positive signals specifically in the IC analysis, including Congenital, familial and genetic disorders (SOC: 10010331, n = 4), Renal and urinary disorders (SOC: 10038359, n = 24), Vascular disorders (SOC: 10047065, n = 29), and Respiratory, thoracic and mediastinal disorders (SOC: 10038738, n = 50).

Of particular note, our analysis detected previously unrecognized safety signals for Skin and subcutaneous tissue disorders (SOC: 10040785, n = 25) and Vascular disorders (SOC: 10047065, n = 29). As these potential risks are absent from the current U.S. prescribing information, they represent uncharacterized aspects of the drug’s safety profile. It is therefore imperative that these signals are rigorously investigated in future dedicated studies to validate their causal relationship with zolbetuximab therapy.

### 3.3 Disproportionality analysis at the preferred term level

Based on a comprehensive analysis of the FAERS database, this study systematically identified and catalogued potential safety signals at the PT level associated with zolbetuximab. The most significant associations are detailed in **Table 3**, with previously unrecognized adverse events specifically marked with an asterisk (*).

**Table 3:** Signal strength of reports of zolbetuximab at the preferred term (PT) level in FAERS database (*The instruction does not mention).

SOC	Preferred terms (PTs)	Zolbetuximab cases reporting PT	ROR (95% two-sided CI)	PRR (χ2)	IC(IC025)	EBGM(EBGM05)
Blood and lymphatic system disorders	Myelosuppression	22	9.18 (6.02-13.98)	9.05 (157.56)	3.18 (2.08)	9.04 (5.93)
Gastrointestinal disorders	Vomiting	84	8.48 (6.80-10.57)	8.06 (521.81)	3.01 (2.41)	8.04 (6.45)
Gastrointestinal disorders	Nausea	193	13.15 (11.30-15.30)	11.58 (1881.80)	3.53 (3.03)	11.55 (9.93)
Gastrointestinal disorders	Retching	6	12.88 (5.77-28.75)	12.83 (65.30)	3.68 (1.65)	12.80 (5.73)
Gastrointestinal disorders	Ascites	34	57.80 (41.05-81.38)	56.50 (1830.73)	5.80 (4.12)	55.79 (39.63)
Gastrointestinal disorders	Salivary hypersecretion	5	21.29 (8.83-51.34)	21.22 (95.90)	4.40 (1.83)	21.12 (8.76)
Gastrointestinal disorders	Gastritis*	9	17.58 (9.12-33.90)	17.48 (139.35)	4.12 (2.14)	17.42 (9.03)
Gastrointestinal disorders	Ileus	7	28.07 (13.33-59.12)	27.94 (180.72)	4.80 (2.28)	27.77 (13.18)
Gastrointestinal disorders	Malignant ascites	3	170.17 (53.64-539.89)	169.83 (484.64)	7.35 (2.32)	163.50 (51.54)
Gastrointestinal disorders	Protein-losing gastroenteropathy*	8	604.48 (288.13-1268.17)	601.25 (4212.95)	9.05 (4.31)	528.49 (251.91)
General disorders and administration site conditions	Oedema peripheral*	17	9.61 (5.96-15.52)	9.52 (129.44)	3.25 (2.01)	9.50 (5.89)
General disorders and administration site conditions	Oedema*	9	7.05 (3.66-13.59)	7.02 (46.41)	2.81 (1.46)	7.01 (3.64)
General disorders and administration site conditions	Performance status decreased	3	32.59 (10.46-101.60)	32.53 (91.01)	5.01 (1.61)	32.30 (10.36)
Hepatobiliary disorders	Hepatic cytolysis	5	5.50 (2.28-13.23)	5.48 (18.30)	2.45 (1.02)	5.47 (2.27)
Infections and infestations	Pneumonia aspiration	5	9.01 (3.74-21.69)	8.98 (35.40)	3.16 (1.31)	8.96 (3.72)
Infections and infestations	Bacteraemia	3	9.38 (3.02-29.17)	9.37 (22.38)	3.22 (1.04)	9.35 (3.01)
Injury, poisoning and procedural complications	Product preparation issue	4	18.62 (6.96-49.77)	18.57 (66.22)	4.21 (1.57)	18.49 (6.92)
Injury, poisoning and procedural complications	Subdural haematoma	3	14.46 (4.65-44.97)	14.43 (37.39)	3.85 (1.24)	14.39 (4.63)
Injury, poisoning and procedural complications	Compression fracture*	3	25.39 (8.15-79.08)	25.34 (69.75)	4.66 (1.49)	25.20 (8.09)
Investigations	Neutrophil count decreased	21	18.49 (12.01-28.47)	18.24 (341.08)	4.18 (2.72)	18.17 (11.80)
Investigations	Platelet count decreased	12	4.82 (2.73-8.51)	4.79 (35.98)	2.26 (1.28)	4.78 (2.71)
Investigations	Blood albumin decreased	25	175.35 (117.20-262.36)	172.43 (4099.19)	7.37 (4.93)	165.91 (110.88)
Investigations	Protein total decreased	3	38.09 (12.21-118.82)	38.01 (107.19)	5.24 (1.68)	37.69 (12.08)
Investigations	Full blood count decreased	4	6.89 (2.58-18.41)	6.88 (20.07)	2.78 (1.04)	6.87 (2.57)
Metabolism and nutrition disorders	Hyperammonaemia	5	44.63 (18.46-107.85)	44.48 (210.38)	5.46 (2.26)	44.04 (18.22)
Metabolism and nutrition disorders	Decreased appetite	70	12.45 (9.79-15.83)	11.92 (700.82)	3.57 (2.81)	11.89 (9.35)
Metabolism and nutrition disorders	Hypoalbuminaemia	27	143.87 (97.74-211.79)	141.29 (3643.41)	7.10 (4.82)	136.89 (92.99)
Metabolism and nutrition disorders	Feeding disorder	10	14.45 (7.75-26.94)	14.36 (123.93)	3.84 (2.06)	14.31 (7.68)
Metabolism and nutrition disorders	Dehydration	22	9.00 (5.91-13.72)	8.88 (153.82)	3.15 (2.07)	8.87 (5.82)
Metabolism and nutrition disorders	Hypokalaemia	6	5.61 (2.51-12.51)	5.59 (22.59)	2.48 (1.11)	5.58 (2.50)
Metabolism and nutrition disorders	Hypophagia	4	6.63 (2.48-17.71)	6.62 (19.05)	2.72 (1.02)	6.61 (2.48)
Neoplasms benign, malignant and unspecified (incl cysts and polyps)	Metastases to bone	4	9.18 (3.44-24.52)	9.16 (29.01)	3.19 (1.20)	9.14 (3.42)
Neoplasms benign, malignant and unspecified (incl cysts and polyps)	Malignant neoplasm progression	35	14.52 (10.38-20.31)	14.20 (428.79)	3.82 (2.73)	14.16 (10.12)
Neoplasms benign, malignant and unspecified (incl cysts and polyps)	Metastases to peritoneum	8	124.32 (61.46-251.50)	123.66 (946.54)	6.91 (3.42)	120.28 (59.46)
Neoplasms benign, malignant and unspecified (incl cysts and polyps)	Metastases to central nervous system	3	9.15 (2.95-28.45)	9.14 (21.70)	3.19 (1.03)	9.12 (2.93)
Neoplasms benign, malignant and unspecified (incl cysts and polyps)	Gastric cancer	5	45.27 (18.73-109.42)	45.12 (213.54)	5.48 (2.27)	44.67 (18.48)
Nervous system disorders	Taste disorder*	5	5.08 (2.11-12.23)	5.07 (16.32)	2.34 (0.97)	5.06 (2.10)
Nervous system disorders	Hypoaesthesia	12	3.79 (2.15-6.69)	3.77 (24.41)	1.91 (1.08)	3.76 (2.13)
Nervous system disorders	Cerebral infarction*	7	16.71 (7.94-35.16)	16.64 (102.52)	4.05 (1.93)	16.58 (7.88)
Renal and urinary disorders	Renal impairment	12	6.16 (3.49-10.87)	6.11 (51.33)	2.61 (1.48)	6.11 (3.46)
Respiratory, thoracic and mediastinal disorders	Interstitial lung disease	10	8.24 (4.42-15.35)	8.19 (63.03)	3.03 (1.63)	8.17 (4.39)
Respiratory, thoracic and mediastinal disorders	Hiccups*	8	46.25 (23.00-92.99)	46.01 (348.58)	5.51 (2.74)	45.54 (22.65)
Respiratory, thoracic and mediastinal disorders	Pneumothorax*	3	8.32 (2.68-25.85)	8.30 (19.24)	3.05 (0.98)	8.29 (2.67)
Skin and subcutaneous tissue disorders	Palmar-plantar erythrodysaesthesia syndrome*	6	10.87 (4.87-24.27)	10.83 (53.45)	3.43 (1.54)	10.81 (4.84)
Vascular disorders	Vascular pain*	4	160.39 (59.06-435.60)	159.96 (609.53)	7.27 (2.68)	154.34 (56.83)

ROR, reporting odds ratio; CI, confidence interval; PRR, proportional reporting ratio; χ2, chi-squared; IC, information component; IC025, the lower limit of 95% CI of the IC; EBGM, empirical Bayesian geometric mean; EBGM05, the lower limit of 95% CI of EBGM.

Notably, the disproportionality analysis revealed several statistically significant and clinically relevant signals, including gastritis, protein-losing gastroenteropathy, oedema peripheral, oedema, compression fracture, taste disorder, cerebral infarction, hiccups, pneumothorax, palmar-plantar erythrodysaesthesia syndrome, and vascular pain.

### 3.4 Time-to-onset of zolbetuximab-related adverse event

**Figure 2:**
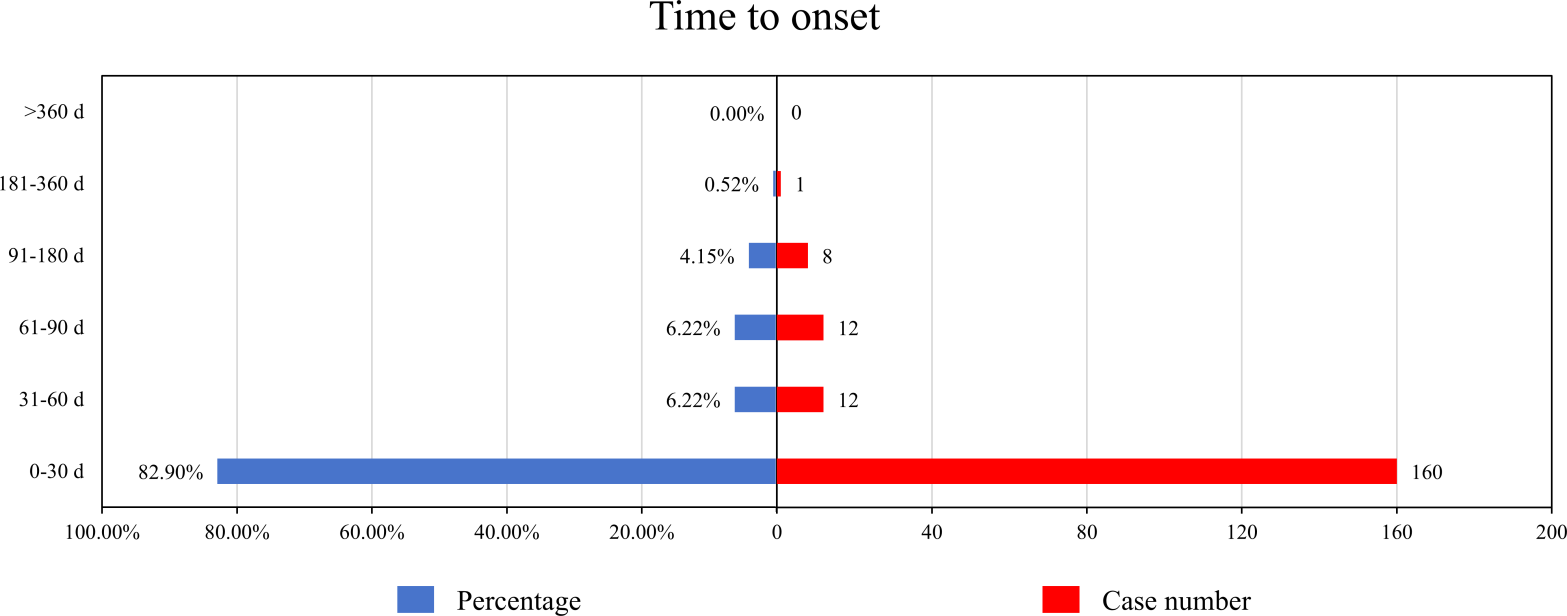
Time-to-onset of zolbetuximab-related AEs.

As shown in **Figure 2** and **Table 1**, following the exclusion of cases with imprecise, unavailable, or incomplete onset date information, a final analysis included 193 adverse events (AEs) with evaluable TTO data, accounting for 28.55% of all reported cases. The median onset time was 0 days, indicating that in many reports the recorded AE date was the same as the zolbetuximab treatment start date. Most AEs (82.90%, n=160) emerged within the initial 30 days following treatment initiation. Conversely, events occurring between 181 and 360 days constituted the smallest subset, representing only 0.52% of the analyzed cases. The frequencies of AEs arising during the 31–60 day (6.22%, n=12), 61–90 day (6.22%, n=12), and 91–180 day (4.15%, n=8) intervals were substantially lower compared to those observed within the first 30 days.

## 4 Discussion

Existing evidence regarding zolbetuximab predominantly originates from controlled trial environments, mechanistic explorations, and narrative literature syntheses, leaving a notable gap in its post-marketing safety characterization based on real-world clinical practice. To address this, we conducted a comprehensive pharmacovigilance investigation employing a large-scale real-world database—FAERS—to systematically evaluate the safety profile of zolbetuximab following its market introduction. The objective of this study was to detect previously unrecognized and clinically meaningful adverse drug reactions, thereby generating novel insights to guide therapeutic decision-making and potentially contribute to future revisions of the approved product labeling.

Disproportionality analyses demonstrated that adverse events associated with zolbetuximab were primarily distributed across several SOCs. The most pronounced safety signals emerged within the domains of metabolism and nutritional disorders, as well as gastrointestinal disorders. Among the most frequently reported and statistically significant PTs were nausea, vomiting, loss of appetite, and weight reduction—findings that are concordant with the recognized safety data outlined in the current FDA-approved prescribing information. Importantly, our evaluation also identified a number of significant potential adverse events not previously documented, some of which may constitute unanticipated safety signals that merit further clinical scrutiny and validation.

Among patients who experienced adverse events related to zolbetuximab, males constituted 65.51% (n = 414/632). This proportion is consistent with epidemiological data indicating that the age-standardized incidence and mortality rates for gastric cancer are over twofold greater in males than in females (25). The age profile of these events also corresponds to the common epidemiology of the disease, with a significant majority (71.04%, n = 395/556) reported in individuals aged over 65 years; the median age was 71 years (IQR: 62–80). Notable geographical variations were observed in reporting frequencies. The highest share of adverse event reports originated from Japan (70.56%), with the United States contributing the second-highest proportion (21.45%). This distribution likely reflects the drug’s development pathway, as zolbetuximab was developed by Astellas Pharma (Tokyo, Japan) and subsequently received U.S. FDA approval as a first-line therapy for locally advanced, unresectable G/GEJ cancer following extensive clinical trials (17).

Gastritis and taste disorder are not mentioned in the FDA prescribing information; however, both clinical studies and case reports have described gastric erosive lesions and gastritis associated with zolbetuximab treatment (26–30). This may result from drug-induced attack on CLDN18.2 expressed on normal gastric mucosal epithelial cells (26, 31). A retrospective analysis revealed that among patients receiving zolbetuximab combined with chemotherapy, up to 89.7% developed gastritis within a median of 7.8 weeks after treatment initiation (27). Endoscopically characteristic findings include mucosal erythema, white exudates, edema, and erosions or ulcers, often presenting diffusely and involving multiple gastric regions (27, 28). Clinically, this form of gastritis is closely associated with loss of appetite, taste disorder and a significant decrease in serum albumin levels (hypoalbuminemia) (27). A deeper understanding of this characteristic adverse reaction can help clinicians better recognize and manage the unique toxicity profile of zolbetuximab.

Although protein-losing enteropathy/gastroenteropathy (PLE), peripheral edema and edema are not listed as adverse reactions in the FDA prescribing information, PLE is a serious complication requiring vigilance during zolbetuximab treatment. Multiple case reports have documented the occurrence of PLE in patients receiving zolbetuximab, manifesting as severe hypoalbuminemia and sometimes accompanied by hypogammaglobulinemia (16, 32, 33). PLE is considered one of the important underlying mechanisms contributing to zolbetuximab-associated edema and hypoalbuminemia (16). Clinical diagnosis is supported by laboratory findings showing a significant decrease in serum albumin and/or immunoglobulins, and confirmed by radionuclide imaging, such as 99mTc-labeled human serum albumin scintigraphy, demonstrating abnormal protein loss from the gastrointestinal tract (33, 34). Regarding its pathophysiology, one case study suggested a possible association with IgA-mediated vasculitis, with IgA deposits detected in the capillary walls of the gastric mucosal interstitium on tissue biopsy (34). This implies that zolbetuximab-induced mucosal injury and increased vascular permeability may underlie the protein loss. During treatment, clinicians should consider the possibility of PLE, especially when patients present with unexplained hypoalbuminemia, and implement appropriate diagnostic and supportive management measures (32, 33).

Vascular pain has been reported in some cases, which may be associated with excessively rapid infusion rates. Strict control of the infusion rate is a core management strategy; approaches such as “stepwise infusion” or “stop-and-go” protocols can be employed, allowing real-time adjustment or temporary interruption of the infusion based on patient tolerance (35, 36). Real-world studies have demonstrated that standardized infusion protocols can significantly reduce the rate of infusion interruptions in subsequent treatment cycles (35).

Adverse reactions such as compression fracture, cerebral infarction, hiccups, pneumothorax, and palmar-plantar erythrodysesthesia syndrome are also not described in the FDA prescribing information, and their potential mechanisms remain unknown. Several case reports have documented their occurrence. With increasing post-marketing exposure and longer follow-up time, further evidence may emerge regarding their potential association with the drug. In clinical practice, both physicians and patients should remain vigilant to the possibility of these adverse reactions.

Large-scale pharmacovigilance studies based on the FAERS database provide a valuable approach for detecting rare adverse events (AEs) and evaluating drug exposure at the population level. However, several limitations of this data source must be acknowledged. First, FAERS is a spontaneous reporting system and is subject to underreporting, duplicate reporting, reporting bias, stimulated reporting after drug approval, and incomplete clinical information. Second, because the total number of exposed patients is unknown, the incidence, prevalence, or absolute risk of zolbetuximab-associated adverse events cannot be calculated. Third, disproportionality analysis identifies statistical reporting associations rather than causal relationships. This limitation is particularly relevant for gastrointestinal events, as zolbetuximab is mainly prescribed for patients with gastric or gastroesophageal junction adenocarcinoma, a population that may already experience nausea, vomiting, anorexia, gastritis-like symptoms, hypoalbuminemia, ascites, or disease-related gastrointestinal complications. In addition, most patients receive zolbetuximab in combination with cytotoxic chemotherapy, including oxaliplatin, capecitabine, and fluorouracil, which themselves are associated with gastrointestinal, hematologic, metabolic, and neurologic toxicities. Therefore, some detected signals may reflect confounding by indication, tumor progression, concomitant treatment, or the underlying disease rather than a direct pharmacologic effect of zolbetuximab. These findings should therefore be interpreted as hypothesis-generating and require validation in prospective studies, electronic health record-based cohorts, clinical trials, or mechanistic investigations. The median TTO of 0 days should be interpreted as same-calendar-day reporting of AE occurrence and treatment initiation. Clinically, this pattern may reflect acute treatment-related events, including infusion-associated symptoms and early gastrointestinal reactions. However, FAERS contains date-level rather than time-level information, and reporting dates may be incomplete or imprecise. Therefore, a 0-day TTO does not necessarily prove immediate pharmacologic causality and should be interpreted cautiously.

## 5 Conclusion

This first large-scale FAERS-based pharmacovigilance analysis of zolbetuximab identified several potential safety signals not currently listed in the prescribing information, including gastritis, protein-losing gastroenteropathy, peripheral edema, compression fracture, taste disorder, cerebral infarction, hiccups, pneumothorax, palmar-plantar erythrodysaesthesia syndrome, and vascular pain. These findings may help refine understanding of the post-marketing safety profile of zolbetuximab and underscore the importance of continued surveillance in clinical practice. However, because FAERS data are based on spontaneous reports and disproportionality analysis detects associations rather than causality, these results should be interpreted as hypothesis-generating and require further validation in well-designed clinical and real-world studies.

## Data Availability

The original contributions presented in the study are included in the article/**Supplementary Material**. Further inquiries can be directed to the corresponding author.
