# Supplementary material for: Safety signals of zolbetuximab in gastric or gastroesophageal junction adenocarcinoma: a comprehensive analysis of the FDA adverse event reporting system (FAERS) data
Source: Front Oncol. 2026 Jul 10;16:1787423. doi: 10.3389/fonc.2026.1787423 (PMC13395747; doi:10.3389/fonc.2026.1787423)
Supplement: Supplementary file 1 [file DataSheet1.docx]

**1 Supplemental Table**

| - 1. **Table S1 Summary of major algorithms used for signal detection.** | | | | |
| --- | --- | --- | --- | --- |
| **Indicator** | | **Equation** | **Criteria** |  |
| ROR | ROR = ad/c/b | | ROR05 > 1, N ≥ 2 |  |
|  | 95CI = eln(ROR)±1.96(1/a+1/b+1/c+1/d)^0.5 | |  |  |
| PRR | PRR = [a/(a+b)]/[c/(c + d)] | | PRR≥2 |  |
| χ2 | χ2 = [(ad-bc)2 (a+b + c + d)]/[(a+b)(c + d)(a+c)(b + d)] | | χ2 ≥ 4, N ≥ 3 |  |
| IC | IC = log2 [a (a+b + c + d)]/[(a+c)(a+b)] | | IC025 > 0 |  |
|  | 95CI = eln(IC)±1.96(1/a+1/b+1/c+1/d)^0.5 | |  |  |
| EBGM | EBGM = a (a+b + c + d)/(a+c)/(a+b) | | EBGM05 > 2, N ≥ 0 |  |
|  | 95CI = eln(EBGM)±1.96(1/a+1/b+1/c+1/d)^0.5 | |  |  |

N, number of adverse event reports; CI, confidence interval; ROR, reporting odds ratio; ROR05, the lower limit of the 95 two-sided CI of the ROR; N, the number of co-occurrences; PRR, proportional reporting ratio; χ2, chi-squared; IC, information component; IC025, the lower limit of the 95 two-sided CI of the IC; EBGM, empirical bayesian geometric mean; EBGM05, the lower 95 two-sided CI of EBGM.

| **1.2 Table S2 Two-by-two contingency table for disproportionality analyses** | | | |
| --- | --- | --- | --- |
|  | **Target adverse event** | **All other adverse events** | **Total** |
| Target drug | a | b | a+b |
| All other drugs | c | d | c+d |
| Total | a+c | b+d | a+b+c+d |
